# Dynamical modelling of secondary metabolism and metabolic switches in *Streptomyces xiamenensis* 318

**DOI:** 10.1098/rsos.190418

**Published:** 2019-04-10

**Authors:** Xiao-Mei Zhu, Xing-Xing Zhang, Run-Tan Cheng, He-Lin Yu, Ruo-Shi Yuan, Xu-Liang Bu, Jun Xu, Ping Ao, Yong-Cong Chen, Min-Juan Xu

**Affiliations:** 1 Key Laboratory of Systems Biomedicine (Ministry of Education), Shanghai Center for Systems Biomedicine, Shanghai Jiao Tong University, 800 Dongchuan Road, Shanghai 200240, People's Republic of China; 2 Shanghai Center for Quantitative Life Sciences and Physics Department, Shanghai University, Shanghai 200444, People's Republic of China; 3 School of Oceanography, State Key Laboratory of Ocean Engineering, Shanghai Jiao Tong University, Shanghai 200240, People's Republic of China

**Keywords:** secondary metabolism, metabolic switch, metabolic modelling, dynamical landscape, systems biology, *Streptomyces*

## Abstract

The production of secondary metabolites, while important for bioengineering purposes, presents a paradox in itself. Though widely existing in plants and bacteria, they have no definite physiological roles. Yet in both native habitats and laboratories, their production appears robust and follows apparent metabolic switches. We show in this work that the enzyme-catalysed process may improve the metabolic stability of the cells. The latter can be responsible for the overall metabolic behaviours such as dynamic metabolic landscape, metabolic switches and robustness, which can in turn affect the genetic formation of the organism in question. Mangrove-derived *Streptomyces xiamenensis* 318, with a relatively compact genome for secondary metabolism, is used as a model organism in our investigation. Integrated studies via kinetic metabolic modelling, transcriptase measurements and metabolic profiling were performed on this strain. Our results demonstrate that the secondary metabolites increase the metabolic fitness of the organism via stabilizing the underlying metabolic network. And the fluxes directing to NADH, NADPH, acetyl-CoA and glutamate provide the key switches for the overall and secondary metabolism. The information may be helpful for improving the xiamenmycin production on the strain.

## 1. Introduction

The secondary metabolites are loosely defined as organic compounds which are not directly involved in the normal physiological activities of the organisms that produce them. Many of these compounds have been actively explored for pharmaceutical and economic purposes [1]. Nevertheless, major fundamental and practical issues remain open. If the secondary metabolites were indeed dispensable or at least non-essential for the organisms, their widespread existence and relatively evolutionary stability within their phylogenetic groups would appear enigmatic. It has been known that many secondary metabolites are triggered by environmental stresses [2] or coincide with physiological changes. The phenomena certainly point to the direction that environmental adaptation may be the primary driving force behind their production [3]. Such a proposal is, we believe, worth further studies conceptually and via well-established quantitative means.

*Streptomyces* sp. is well known for producing novel secondary metabolites. The dynamic transcriptional and translational landscape on the strain *S. coelicolor* A3(2) was obtained previously [4], though a theoretical model for metabolic switches that orchestrate the metabolism from its big genome size (*ca* 8 Mb) remained elusive. *Streptomyces xiamenensis* 318, selected for the present study, has a relatively compact genome (*ca* 5.96 Mb) among the *Streptomyces* species, producing two types of secondary metabolites. The gene cluster responsible for producing polycyclic tetramate macrolactams (PTMs) is located at the centre of the genome, whereas xiamenmycin biosynthetic gene cluster sits in a dispensable genomic island close to one of the arm regions in the linear chromosome [5]. Furthermore, the gene cluster encoding PTMs is universally present in many *Streptomyces* spp. as well as in other bacterial genera [6]. The specific genomic location and its apparent evolution conservation point to some important yet unknown physiological role of PTMs. By contrast, the biosynthetic gene cluster for xiamenmycin could be found only in a few species, such as *S. xiamenensis* and *S. himastatinicus.* It presumably spread via horizontal gene transfer [5]. The presence of the unique xiamenmycin raises questions on its association with the adaptation to the bacterial producer's habitat of mangrove.

To explore the underlying physiological mechanism for biosynthesis of the secondary metabolites, we carried out in this work integrated studies which include dynamic metabolic modelling, transcriptome-level measurements, genetic and metabolic experiments on *S. xiamenensis* 318. Our results demonstrate from various perspectives that the production of the secondary metabolites, a typical response to environmental stresses, does indeed increase the metabolic fitness of the organism. It thus strongly supports the environmental adaptation hypothesis mentioned earlier.

## 2. Results

### 2.1. Metabolic modelling of *Streptomyces xiamenensis* 318

**Figure 1.**
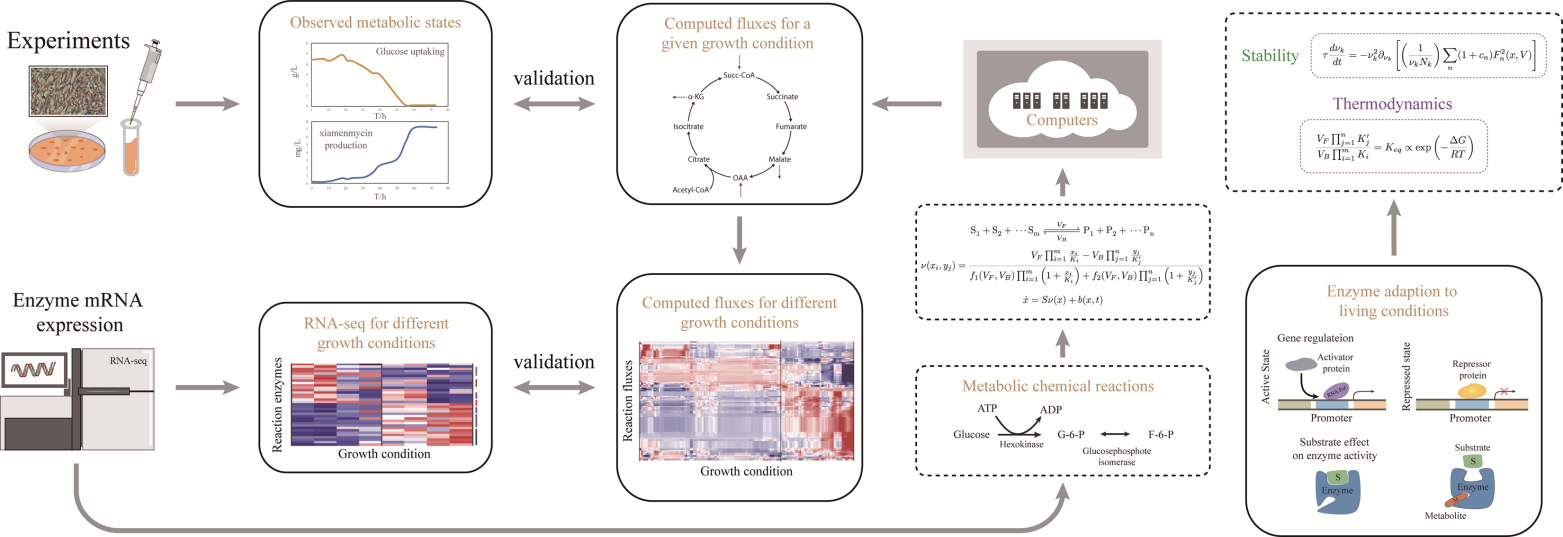
Schematics of large-scale metabolic network construction and modelling for *S. xiamenensis* 318. Metabolic profiles in batch cultures were measured at different times. Together with gene expression measurements, the active chemical reactions responsible for the central metabolism and the secondary metabolite production were identified. Under dynamical stability regulations, metabolic reaction equations were established for different nutrient conditions and specific metabolic biomass output requirements. Calculated metabolic fluxes demonstrated clear-cut metabolic states that correspond to the measured gene expressions of key metabolic enzymes.

As mentioned above, metabolic stability can heavily influence the production of the secondary metabolites. We intend to search for more generic principles on the establishment of metabolic patterns. The approaches employed for the study are summarized in figure 1. Upon self-regulations towards better dynamical stability, the metabolic network appears to be well ordered. When it is simulated under different nutrient intakes and metabolic biomass output conditions, the central metabolism is found to be protected from variations of peripheral metabolic fluxes in most cases. More technical details on the modelling methodology can be found in §4.4.

### 2.2. Metabolic profiling of *Streptomyces xiamenensis* 318 under different growth phases

*Streptomyces* are filamentous Gram-positive soil-dwelling bacteria. Their metabolism and regulation are strongly related to the soil environments which are typically carbon-rich but nitrogen- and phosphate-poor [7]. In our case, *S. xiamenensis* 318 was isolated from estuarine sediment [8], which is indeed a carbon-rich but nitrogen-limited ecosystem [7].

**Figure 2.**
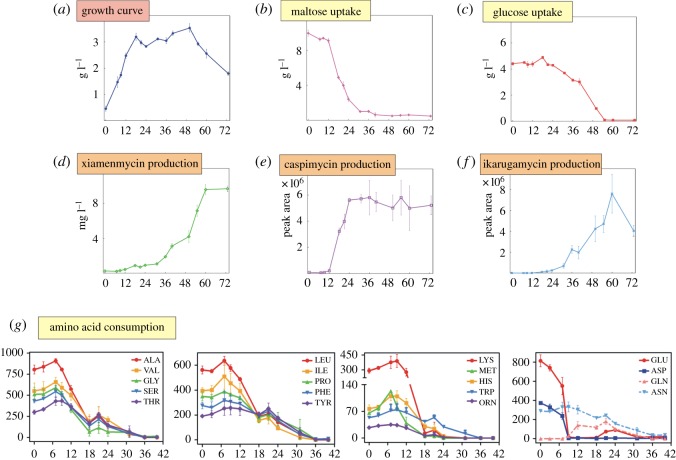
Time-series characterization of carbon and nitrogen sources consumption and the secondary metabolite production in *S. xiamenensis* 318. The figure shows the increase of biomass (*a*), the consumption of maltose (*b*), glucose (*c*) and amino acids (*g*) as well as the production of xiamenmycin (*d*), capsimycin (*e*) and ikarugamycin (*f*) during a complex medium cultivation in batch fermentations. The concentration is in a relative scale.

In the batch culture, the earliest nutrition change during an exponential growth phase was depletion of amino acids. Similar to *S. coelicolor* [9], glutamate was a preferred nutrient for *S. xiamenensis* 318, and most of the amino acids were exhausted before glucose utilization started (figure 2). Glutamate (GLU) and aspartate (ASP) were first depleted in the rapid growth phase. The amino acid levels in complex media tended to increase during the early rapid growth phase and then were depleted just before the biomass reached its maximum. Since the complex culture medium contained certain amounts of peptides and proteins, the increase could probably be attributed to the peptide or protein breakdown. It can be inferred that nitrogen starvation had been experienced during the late exponential growth phase. We also observed that maltose was the preferred carbon source of *S. xiamenensis* 318 to support the biomass accumulation, and its rapid consumption was observed after the depletion of amino acids.

In our previous work, we had identified two major secondary metabolites that were produced in *S. xiamenensis* 318, i.e. the benzopyran derivatives or xiamenmycins, and a group of PTM compounds (capsimycin and ikarugamycin) [10]. During the lifespan of a batch culture on the complex medium, *S. xiamenensis* 318 first underwent a metabolic switch from an exponential growth phase to a stationary phase producing both PTMs and xiamenmycin A in a time series (figure 2*c*). A highly oxidized PTM compound, capsimycin, was detected first and its concentration reached the steady state in the middle of the stationary phase together with the maximal consumption rate on maltose. Xiamenmycin A was produced in the late stage of the lifespan of *S. xiamenensis* 318.

### 2.3. Transcriptomic profiling of *Streptomyces xiamenensis* under different growth phases

To explore the relationship between the primary and the secondary metabolism, the global gene expression levels of strain 318 from the early to the late stationary phase were compared by RNAseq analysis at 16, 24, 36 and 72 h after a batch culture started.

When the strain 318 reached the stationary phase, the transcription levels of the genes involved in glycine, serine and threonine metabolism, glyoxylate and dicarboxylate metabolism, as well as terpenoid backbone biosynthesis were upregulated at 16, 24, 36 and 72 h (see electronic supplementary material, figure S1). The expression levels of the genes involved in nitrogen metabolism, citrate cycle (TCA cycle) and alanine, aspartate and glutamate metabolism were downregulated (electronic supplementary material, figure S2). The transcription levels of the genes related to glycolysis/gluconeogenesis, pyrimidine, starch and sucrose, amino acid biosynthesis were downregulated at first, but recovered afterwards until 36 h, followed by another drop at the late stationary phase. Similarly, the expression levels of the genes involved in arginine and proline metabolism, together with the biosynthesis of PTMs, were found to change in the same pattern. According to the RNAseq data, the metabolisms of maltose, pyruvate and pyrimidine were also active in the metabolic network of strain 318, reflecting some global changes in the major metabolic pathways when the secondary metabolism was active.

The essential metabolic network supporting xiamenmycin biosynthesis was established from the genomic study of *S. xiamenensis* 318 in our previous work [5]. To explore the relationship between xiamenmycin biosynthesis and the central metabolism during different growth phases, the transcriptomes of *S. xiamenensis* were compared under the time series. Differentially expressed genes (DEGs) were identified at selected time points at 16, 36 and 72 h (electronic supplementary material, figures S1 and S2).

The biosynthetic pathway of xiamenmycin was encoded by a gene cluster *ximA-E* [11]. The expression of *ximA-E* genes started at 16 h, continued to 36 h and reached the maximal expression level at 72 h, in accordance with the detection of the maximal concentration of xiamenmycin. We observed the co-occurrence of the onset of xiamenmycin biosynthesis with the switching of carbon source from maltose to glucose. The expression level of the gene cluster *xim* kept getting upregulated under nutrient deficiency.

The common skeleton of PTMs originates from one ornithine and two 12-carbon chains [12]. A gene cluster that consists of *ikaABC* was proved to be responsible for encoding a hybrid of PKS and NRPS pathway in the biosynthesis of PTMs [12,13]. In accordance with the observation that the capsimycins were rapidly produced at 12 h, the *ikaABC* genes were highly expressed at first, then were downregulated at 16 to 24 h, but recovered from 24 to 36 h. DEGs were identified at selected time points 16, 24 and 36 h to explore the association between the production of PTMs and the metabolic pathway activities.

It was reported that glutamate depletion was also the onset for the conversion of the nutrient intake to arginine and fumarate via urea cycle [14]. There are two pathways converting glutamate to ornithine. The first pathway is the condensation of glutamate with acetyl-CoA, yielding *N*-acetyl-glutamate, the initial compound for ornithine and arginine synthesis and an activator of carbamoyl synthesis [15]. Alternatively, glutamate could get converted to gluatamate-5-semialdehyde after phophorylation and reduction before entering the urea cycle, which is also the degradation pathway for intermediates in the urea cycle [15]. We observed that glutamate, one of the major amino acid constituents in GYM medium, was consumed rather rapidly, and the two pathways converting glutamate to ornithine were differently expressed during the PTM production in *S. xiamenensis* 318 strain.

Glutamate can be decarboxylated to 4-aminobutyrate (GABA) and it eventually degrades to succinate [15]. It is well known that the flux of urea cycle increases in animals and bacteria during protein-rich diets [15]. In our measurement, such association also occurred, which might be an alternative explanation for the early appearance of capsimycins as the secondary metabolites. We concluded that the production of PTM compounds was most likely initiated by glutamate depletion.

### 2.4. Two distinct classes of metabolic steady states with variant flux distributions

In a series of previous works, a method to model the dynamics of a large metabolic network has been developed [16–18]. Central to the modelling method is the underlying methodology that a metabolic network can be self-regulated to reach steady and stable states. With the regulation, the fitness level or the robustness of each steady state can be accurately estimated and compared. In natural habitats, bacteria must adapt according to nutrient availability and other environmental conditions. In the metabolic modelling, these conditions can be simulated by the availability of nutrient intakes and changes in the metabolites/biomass outputs [16–18]. For instance, at high temperature lipid turnover increases due to oxidation and membrane fluidity changes [19]. Such heat-shock stress can be modelled by increasing the output production of acetyl-CoA, NADPH and ATP, as the latter are required for lipid synthesis. We have modelled a variety of nutrient and other stress circumstances by changing nutrient intakes and boundary conditions on the simulated metabolic network. These conditions aimed to account for the availability of carbon sources and amino acids, as well as ATP and NADH supplies.

**Figure 3.**
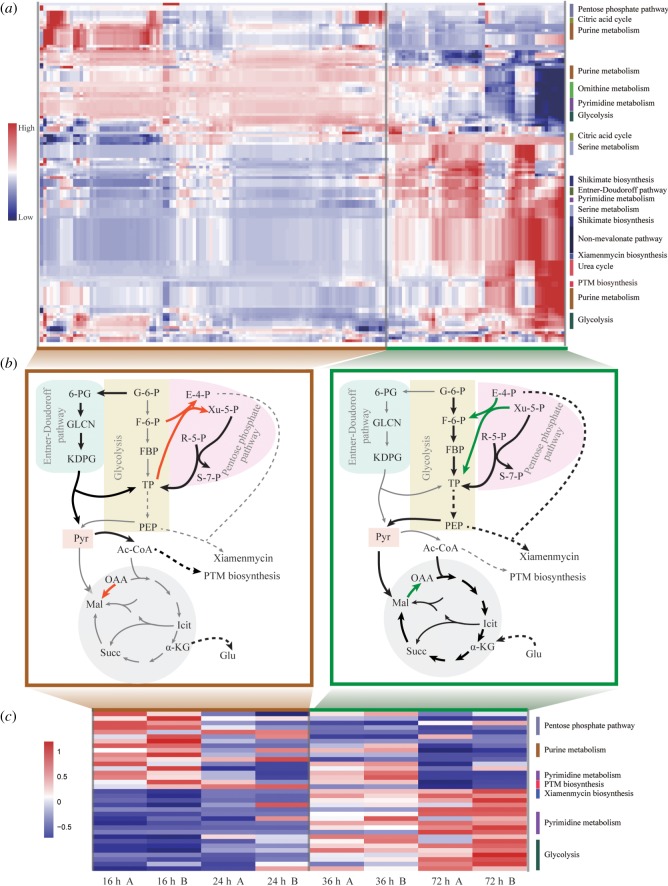
Comparison between computed results and RNAseq data on two groups of metabolic states. (*a*) Modelled metabolic fluxes, normalized for each reaction for *S. xiamenensis* 318 under different nutrient intakes and biomass output conditions. See also electronic supplementary material, figure S4. (*b*) Schematic graph showing two distinct metabolic categorizations with different flux distributions. Red and black/green represent increased/decreased fluxes. (*c*) RNAseq data measured at different time, i.e. 16, 24, 36 and 72 h, in double columns represented by A and B. More details can be found in electronic supplementary material, figure S5.

The metabolic switching behaviour on glycolysis and TCA fluxes is well known biologically [20–22], for example the metabolic switch from exponential growth to antibiotic production in *S. coelicolor* [9,23]. In our case, the calculated landscape of metabolic fluxes can be grossly divided into two different groups. They are represented by normalized high/low glycolysis versus TCA cycle fluxes or xiamenmycin versus PTM biosynthesis fluxes in figure 3 and electronic supplementary material, figure S4.

### 2.5. Metabolic pattern establishment by robustness and switches

**Figure 4.**
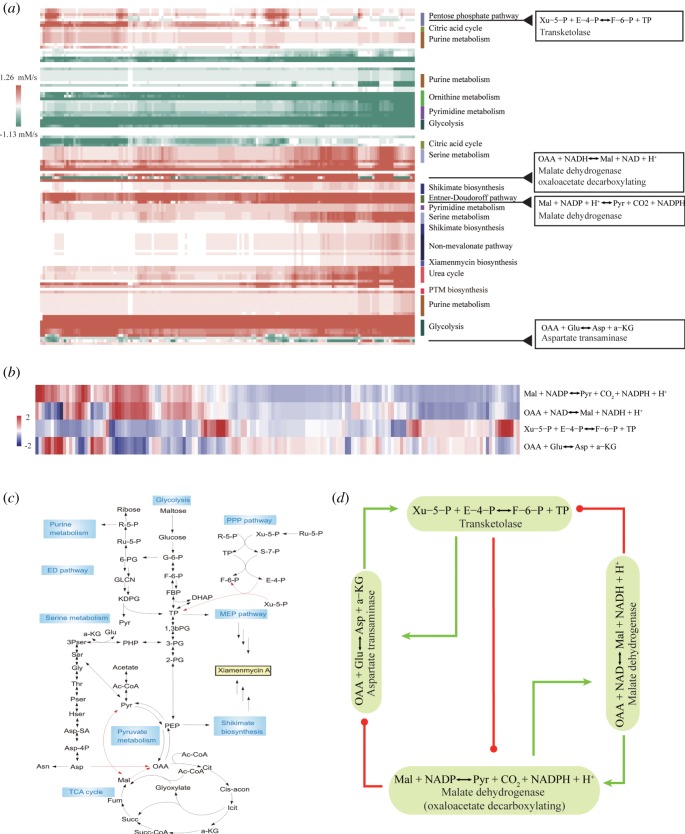
Identification of the metabolic switches by modelling analysis. (*a*) Modelled metabolic fluxes shown in absolute values for *S. xiamenensis* 318 under different nutrient intakes and metabolic/biomass output conditions. See also electronic supplementary material, figures S6 and S7. Among them, five reactions show significant flux changes while the others are ‘buffered’, i.e. their flux directions and values stay relatively unchanged. (*b*) Among the five reactions showing significant flux changes, four are in the central metabolism and their detailed values are presented. (*c*) Enlarged core part of the whole metabolic network from electronic supplementary material, figure S3. (*d*) The flux distribution can be obtained by an effective ‘enzyme’ interaction or regulatory network based on the selected five reactions.

The simulated metabolism for different nutrient intakes and biomass output demands is highly structured (figure 4; electronic supplementary material, figure S6–S7). Since mass conservation must be followed on a metabolic network, the ability to adjust to a variety of nutrient intakes and metabolic outputs while maintaining a majority of fluxes unaffected implies the possible existence of a special mechanism for ‘metabolic control’. In figure 4, fluxes are presented without normalization. It is clear that most of the fluxes undergo no significant change in either direction or absolute value except for five of them. The latter include four fluxes located at the central metabolism (electronic supplementary material, figure S7). These outcomes may be interpreted as the results of a buffering effect provided by these few reactions. In an analogy, they are the reversible traffic lanes in a city full of one-way traffic. We refer to them as the ‘key switches’ in figure 1.

For a large metabolic network, this is a surprisingly small number of ‘buffers’. Among these four reactions, three connect to the TCA cycle, while one feeds into the pentose phosphate pathway (PPP). Our conjecture is that by maintaining these two metabolic units (TCA and PPP), the central metabolism can stay shielded from environmental impacts. The connections of metabolites involved in these four reactions are shown in figure 4. Indeed, these metabolites form two connecting clusters which actively participate in the TCA cycle and the pentose phosphate pathway. The buffering effect can be illustrated with the example of Xu-5-*P* + *E*-4-*P* ↔ F-6-*P* + TP, which increases/decreases its flux in the pentose phosphate pathway, depending on the reaction directions.

To confirm the observation that these four reactions effectively control the overall metabolism, we systematically varied the input and output fluxes of these metabolites in our model, e.g. the glutamate intake and the acetyl-CoA output fluxes. We found that only fluxes of four metabolites, NADH, NADPH, acetyl-CoA and glutamate, can affect the overall metabolism to a degree that the types of metabolic states, as defined in figures 1 and 4, are switched. Equivalently, only metabolic changes of these four metabolites can switch on/off the production of the secondary metabolite xiamenmycin. These four metabolites are directly or immediately connected to the four key reactions, as shown in figures 1 and 4. Note that the existence of such switches suggests that any approach to metabolite engineering, besides focusing on enzymes directly involved in the biosynthetic pathway of the secondary metabolites (e.g. xiamenmycin production), will need to take into account scenarios affecting fluxes of NADH, NADPH, acetyl-CoA and glutamate.

Metabolic robustness is maintained biologically, at least in part, due to tight regulations of nutrient transports and enzyme concentrations via complex biological processes including transcriptional regulations. One sees no explicit biological regulations integrated into our model at first sight. But most crucially, central and built-in to our modelling method is a self-regulation algorithm that drives a metabolic network towards steady and stable states. The regulation, a dynamical algorithm derived formally from a stochastic dynamics that deals specifically with environmental fluctuations, has been introduced to play the similar roles to the metabolic network *in silico* as the biological processes to the real cells. Within our modelling platform, the fitness level or the robustness of each steady state can be accurately estimated and compared. In this regard, the requirement on the metabolic stability has been formulated into the said dynamical algorithm as a manifest of the real-world biological regulations [16]. As a metabolic network is intrinsically different from a gene regulation network due to the mass conservation constraint, it is intriguing to uncover how robustness in metabolic fluxes can be achieved under varying nutrient intakes and metabolic demands (figure 4*d*).

### 2.6. The secondary metabolites and metabolic network stability

To investigate the possible roles of biosynthesis of the secondary metabolites to global metabolism, the initial values of reaction rates in the dynamical reaction equations were varied (cf. §4.4 for technical details). The set of parameters supporting steady states on a metabolic network are not unique [16]. Different sets of parameters have their own dynamical characteristics. They correspond to biologically steady states with different amounts of total enzymes. A quantitative definition for the stability of a steady state on the metabolic network can be characterized by the so-called relaxation time (RT) of the state. RT is an estimation on the speed that the network reacts to small fluctuations from a given steady state.

Typically, a more efficient network, i.e. with less nutrient intake for a given set of biomass output and less waste on futile cycles, is usually found to be less viable due to longer RT and a wider spread of metabolite concentrations. Hence the shorter the RT, the more stable or robust the steady state. We found that for the chemical reaction rates obtained with different initial values, RT decreases with the increase on the secondary metabolite production, as shown in electronic supplementary material, table S1.

To further demonstrate the importance of the secondary metabolites to metabolic network stability, we then performed simulations on ‘knockout’ strains. When both PTMs and xiamenmycin production fluxes were eliminated, the RT values increased significantly. The result is presented in electronic supplementary material, table S2. We therefore concluded that the secondary metabolite production is favoured for improving the metabolic stability or enhancing the metabolic ‘fitness’.

**Figure 5.**
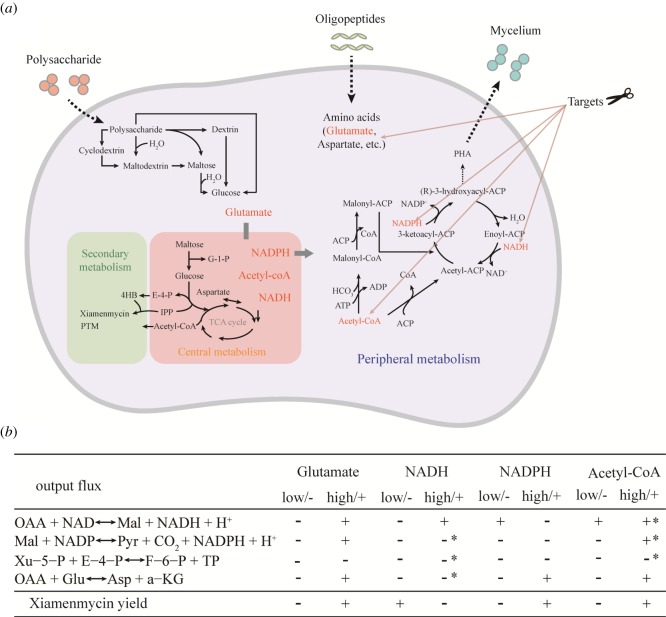
Possible targets for metabolic engineering based on our metabolic modelling. (*a*) The total metabolism for *S. xiamenensis* 318, containing the central metabolism and the secondary metabolism (marked in pink and green), as well as other metabolic pathways not included in the model (under peripheral metabolism). The flux exchanges are referred to as nutrient intakes (input) and metabolic demands (output). The table in (*b*) gives the details. The symbol +/− represents increased fluxes (greater than 0.1) or decreased fluxes (less than 0.1). The input/output flux changes may be achieved by modifying the peripheral metabolism.

These *in silico* results are in agreement with the proposed association of the secondary metabolites to the adaptation under stressed environments. Our modelling analysis also reveals that the two types of the secondary metabolites in *S. xiamenensis* 318 differ in their contributions to the stress reduction. If the demands for acetyl-CoA and NADPH increased due to stress, the production of xiamenmycin would be favoured. But in most other cases, PTM production dominates, which is consistent with the experimental observations (figure 1*d,e*). Therefore, any attempt for bioengineering improvement on the xiamenmycin production will need to take the above factors into account. A schematic presentation of the relationship between the key reactions, the fluxes of the key metabolites and the production of xiamenmycin, is given in figure 5.

Finally, there is an equivalent description of our results which may be worth further investigation, possibly linking metabolism to genome structuring. Since only four reactions in the central metabolism show qualitative differences in our modelling, a metabolic steady state (figure 3) can be viewed as one set of combinations of these four reactions. When we assign values to these reactions (starting in high flux from left to right), the regulatory network between these reactions as seen in figure 4*d*, denoted by their enzyme names, reproduces the two configurations in figure 3 as the network attractors. This can be easily verified for the reaction catalysed by malic enzyme, and malate dehydrogenases are in the same direction and are opposite to that of aspartate transaminase and transketolase in figure 4*d*. In other words, the ‘genetic switching circuit’ represented by these four enzymes will lead ‘naturally’ to the metabolic landscape reported in figure 3 if the circuit ‘logics’ are implemented literally [24]. The finding portraits a simple picture for this particular metabolic network: the metabolism can be regulated by the simple ‘circuit’ to achieve the desired metabolic stability. Is the regulatory description only a mathematical equivalence, or is this particular bacterium genome indeed constructed under such an organization principle?

## 3. Discussion

The secondary metabolites belong to natural products of low molecular weight. They are restricted in taxonomic distribution, specific to individual species, and synthesized for a finite period of time when cells have stopped dividing. Their probable physiological function is often defined as convenient disposal packages of excess primary substances [25]. However, such a role of waste disposal as the sole reason for the production of the secondary metabolites cannot be ultimately satisfactory. For instance, if fitness were not involved, genetic mutations and horizontal gene transfers could have led to a multitude of non-toxic, well-mixed waste packages for each species.

The coincidence of the initiation of morphological differentiation and the biosynthesis of the secondary metabolites indicates that the biological activity of the small molecules at high concentration may protect their producer when the latter is vulnerable during the transition phase of development [26]. As revealed by genome-scale gene function annotation, microorganisms represented by *Streptomyces* are bestowed with dozens of putative gene clusters encoding the secondary metabolites [27], which add contributions to the chemical world of the producer, i.e. the parvome [28]. In natural habitats, the small molecules are always produced at sub-inhibitory concentration [29] and can serve as chemical signals that regulate the gene expression of neighbouring organisms [30]. From an ecological perspective, the secondary metabolism can play essential roles in social or predatory occasions in the microbial community [3].

Nevertheless, it is hard to comprehend why *S. xiamenensis* 318, as a monogenic population started from a pure culture, produces two types of the secondary metabolites orderly in batch culture. Therefore, the physiological role that stabilizes metabolism becomes an appealing alternative. The secondary metabolite knockout strains usually have no impairment in bacterial growth. Two different scenarios may account for such observations. Either there is a redundancy in the secondary metabolite functions or, indeed, they are simply wastes packed for disposal. While it is difficult to obtain knockout strains clear of all the possible secondary metabolites, the mechanistic study may be conducted *in silico*. Using such ‘dry experiments’, we found that the knockout model on both the secondary metabolites is seriously impaired in its dynamical stability as quantified by a significant increase in RT, implying non-dispensable, yet partially redundant roles for the secondary metabolites.

Metabolic fluxes for *S. xiamenensis* 318 calculated under different nutrient intakes were well organized. Only four central metabolic fluxes changed significantly in an absolute scale, suggesting that they may act as the key reactions or switches. The fluxes of these reactions correspond to two topological classes. Modifying the input and output fluxes of the four metabolites in the network may effectively switch between these two classes. These four flux distributions may be considered as the results of an effective regulatory network which has an adaptive network landscape of two attractive basins.

The implication of our results to bioengineering is less affirmative. The production in the native strain under its natural habitat is, presumably, optimized by evolution. Take xiamenmycin as an example. Since it is a product most likely favoured under acetyl-CoA and NADPH stress, a simple increase in its synthesis enzyme concentration does not lead to significant extra production. Nevertheless, one can envision a totally different strategy: it is possible to use certain heterologous expression of the biosynthetic gene cluster in another optimized bacterial chassis host. In a recent publication, we demonstrated that xiamenmycin B, an intermediate precursor of xiamenmycin A and one that does not incorporate threonine into the benzopyran core skeleton, could be synthesized in *E. coli*. [31]. Whether the surrogate *E. coli* host is more adaptable to the acetyl-CoA and NADPH stress than the natural *Streptomyces* host merits further study.

Quantitative method can provide a convenient and transparent analysis tool for difficult experimental conditions. A strain with no secondary metabolite production is difficult to cultivate by genetic manipulation, probably owing to the reduced metabolic fitness as predicted by our *in silico* modelling. Another factor causing the difficulty can be the intriguing ‘buffering’ effect on the internal metabolic fluxes. For example, when we increased the output flux of an arbitrary metabolite, such as alanine, malate, or even ATP, the internal flux distributions in PPP or TCA were qualitatively undisturbed. However, the underlying metabolic states could switch their types in association with the production of the secondary metabolites. It is plausible that these secondary metabolites might have evolved to be one of the factors regulating the central metabolism.

Traditionally, static analyses of metabolic network based on the flux balance analysis (FBA) were used in the literature to simulate the growth and production of the secondary metabolites under various physiological conditions, including impacts of knockout on studied strains, e.g. *Streptomyces clavuligerus* [32]. By contrast, we employed a dynamical modelling technique for the metabolic network. The dynamical approach is a close resemblance of the real chemical reaction network and is more transparent on the constraints imposed. Ideally, the difference in fitness should be measured in co-culturing experiment on the wild-type and the mutant strains. Our modelling study offers a convenient ‘dry way’ out, a quantitative access to the fitness level of a given steady state. Thus it is especially suitable for robustness study on metabolism.

## 4. Methods

### 4.1. Strains, culture conditions and growth experiments

The *S. xiamenensis* 318 was isolated from a mangrove sediment sample which was determined to be *S. xiamenensis* by 16S rRNA gene sequence analysis. Fresh spores of strain 318 formed by cultivation on SFM plates (soybean flour 20 g l^−1^, d-mannitol 20 g l^−1^, agar 20 g l^−1^, pH = 7.2) were incubated at 30°C for 7 days and were then harvested and suspended in the appropriate amount of sterilized and distilled water. They were filtered to remove medium fragments and were then preserved in the 20% (v/v) glycerol tubes (2 ml).

Batch experiments were performed in 250 ml Erlenmeyer flasks containing 50 ml culture medium. *Streptomyces xiamenensis* 318 started from spore suspensions was grown in tryptone soy broth (TSB) medium (Oxoid) at 30°C for 40 h in a rotary shaker at 200 r.p.m. as seed cultures. Subsequently, the seed cultures were transferred into 100 ml GYM medium (ISP2 medium, glucose 4 g l^−1^, yeast extract 4 g l^−1^, malt extract 10 g l^−1^, pH = 7.2) at 3% (v/v) in 500 ml flasks and cultured at the same conditions as the seed cultures.

For the measurement of growth of *S. xiamenensis* in media without insoluble particles, we used dry weight measurements. Each culture sampling at a certain time was centrifuged at 8000 r.p.m. for 10 min using a pre-dried and pre-weighed tube. The residue was dried at 95°C overnight after being washed twice with sterilized and distilled water, while the supernatant remained to be extracted. After cooling at room temperature without exposure to moisture, the dry weight of residual cultures was measured as the difference between the weight of tube before and after centrifugation. All dry weight measurements were carried out in triplicates.

### 4.2. Measurements of extracellular glucose, maltose and free amino acid concentrations

Concentrations of glucose and maltose were determined spectrophotometrically by using the Glucose (HK) Assay Kit (GAHK-20, Sigma-Aldrich) and Maltose Assay Kit (MAK019, Sigma-Aldrich), following the manufacturer's instructions. Amino acids were measured using the EZ:faast™ GC-MS free amino acid analysis kit of Phenomenex which was previously used in amino acid uptake profiling of *S. lividans* [33]. Derivatization and preparation of samples were following the protocol, and amino acids were measured by GC-MS (QP-2010 Ultra, Shimadzu). The Zebron ZB-AAA capillary column (10 m × 0.25 mm) supplied with the test kit was used. Helium was used as the carrier gas at a flow rate of 1.2 ml min^−1^. The GC-MS conditions were as follows: the injection temperature, 300°C; the inlet line temperature, 320°C; the ion source temperature, 240°C; initial oven temperature, 110°C; held for 0.5 min then increased to 310°C at 30°C min^−1^, held for 0.5 min. The scan range was 45–450 (3.5 scans s^−1^). Under these conditions, a 1 µl sample was injected in a split (1 : 15) mode.

### 4.3. Quantitative measurements of xiamenmycin and polycyclic tetramate macrolactams

The supernatants from centrifuging GYM broth were extracted three times at room temperature with equal volumes of a solvent mixture of ethyl acetate : methanol (85 : 15, v : v). The supernatant was combined and concentrated under vacuum at 40°C to remove the organic phase. Each crude extract was subsequently re-dissolved in the same volume of HPLC grade methanol (4 ml). The samples were analysed by HPLC (Prominence, Shimadzu) using a ZORBAX Extend-C18 column (4.6 × 150 mm, 5 µm, Agilent) and monitored by UV detection at 254 nm. The solvent system of H_2_O (A) and methanol (B) was used as the mobile phase in the following linear gradient programme: 0 min 20% B, 5 min 20% B, 30 min 100% B, 35 min 100% B, 36 min 20% B, 40 min 20% B, at a flow rate of 1 ml min^−1^. The xiamenmycin A obtained by our group was used as the standard reference.

### 4.4. Network Lyapunov function and self-regulated metabolic modelling

For kinetic modelling of metabolism, a large number of enzymatic parameters have traditionally been a major obstacle. But, the full knowledge of the mechanistic reaction rates is often not required for characterizing the behaviour of an organism. This is because physiologically metabolite concentrations are usually restricted to a rather narrow subspace from the whole range [16–18]. Since most biological reactions are catalysed by enzymes, one can construct the kinetic model based on a generic enzymatic rate equation. Central to our modelling methodology is a self-regulation algorithm that resembles real biological processes in maintaining metabolic stability and requires only a small set of boundary conditions [16–18]. We present below a brief summary of the methodology.

Take a general chemical reaction

4.1 $$A_{1}+A_{2}+\ldots +A_{m}\overset{V_{B},V_{F}}{⟷}P_{1}+P_{2}+\ldots +P_{n},$$

where each *A_i_* or *P_j_* can be the same substrate or product as the previous one. Equation (4.1) specifies the stoichiometry with an implicit enzyme(s) that applies to both directions of the reaction. Our simulation employs a generic enzymatic rate equation of the form [16,17]

4.2 $$\begin{array}{rl} & u([A_{i}],[P_{j}])=\frac{V_{F}\prod_{i=1}^{m}[A_{i}]/K_{i}-V_{B}\prod_{\;j=1}^{n}[P_{j}]/{K^{\prime }}_{j}}{\;f_{1}(V_{F},V_{B})\prod_{i=1}^{m}\left(1+[A_{i}]/K_{i}\right)+f_{2}(V_{F},V_{B})\prod_{\;j=1}^{n}\left(1+[P_{j}]/{K^{\prime }}_{j}\right)}. \end{array}$$

where *V*_F_/*V*_B_ is the forward/backward peak reaction rate and the bracket [*A_i_*]/[*P_j_*] refers to the concentration of the substrate/product with $K_{i}/K_{j}^{\prime }$ being its Michaelis–Menten constant on the reaction. The two slowly varying normalization factors *f*_1_ and *f*_2_ satisfy

4.3 $$f_{1}(V_{F},V_{B})+f_{2}(V_{F},V_{B})=1,$$

4.4 $$f_{1}(V_{F}=0,V_{B})=0$$

4.5 $$\;\mathrm{and}\;f_{2}(V_{F},V_{B}=0)=0.$$

Accordingly, a metabolic network with *N* metabolites and *M* reactions whose dynamics can be described by

4.6 $$\frac{dx(t)}{dt}=S\cdot u=f(x,\;V).$$

where **x**(*t*) is an *N* × 1 vector for the metabolite concentrations, **u** is an *M* × 1 vector for the reactions, *S* is the stoichiometric matrix connecting the reactions to the metabolites and **V** denotes the parameter set for all the reactions (e.g. {*V*_F_, *V*_B_}).

A dynamical system under equation (4.6) is usually unstable as some metabolites will quickly accumulate and some others diminish. We therefore need to incorporate some biological aspects into our model. A living cell can adjust its parameters and maintains the metabolic stability under fluctuations of concentrations in both metabolites and enzymes (reaction rates). To find out how the enzymatic parameters should be regulated in order to achieve stability across the network, we can add random noises to equation (4.6) and study the stochastic processes that can help in achieving network stability against the fluctuations.

Following the formal mathematical analysis developed previously [16,34–37], one can always define quite generally a monotonically decreasing Lyapunov function φ(x(t),V) for equation (4.6). The construction of the Lyapunov function is closely related to the diffusion matrix of the stochastic fluctuations. One can further associate a non-negative cost function ψ(x(t),V) to the change of the Lyapunov function in time,

4.7 $${\frac{d\varphi }{dt}|}_{V=\mathrm{fixed}}=-\psi (x,\;V)=-f^{T}(x,\;V)\cdot \Sigma (x,V)\cdot f(x,\;V),$$

where the superscript *T* denotes the matrix transpose and Σ(x, V) is a *N* × *N* positive-definite matrix. Therefore, equation (4.6) may be be stabilized by a regulation process that minimizes ψ(x, V) (on given **x**), which can take the generic form

4.8 $$W(x,\;V)\cdot \frac{dV}{dt}=-\nabla_{V}\;\psi (x,\;V),$$

where W(x, V) can be a positive-definite modulation matrix for computational purpose.

The postulation that equation (4.8) can resemble real, albeit complex biological regulations as well as approximations and choices for Σ(x, V) and W(x, V) was discussed in detail in the previous work [16]. One can first apply the regulation procedure to *V_k_* ∈ {*V*_F_, *V*_B_} which reads,

4.9 $$\frac{dV_{k}}{dt}=-V_{k}^{2}\frac{\partial }{\partial V_{k}}\left[\left(\frac{1}{V_{k}N_{k}}\right)\sum_{n}\left(1+c_{n})f_{n}^{2}(x,\;V\right)\right],$$

where the summation is over the *N_k_* number of metabolites affected by *V_k_*, and *c_n_* is the number of carbon atoms carried by the *n*-th metabolite. The thermodynamic constraint for each reaction

4.10 $$\frac{V_{F}\prod_{\;j=1}^{n}K_{j}^{\prime }}{V_{B}\prod_{i=1}^{m}K_{i}}=K_{\mathrm{eq}}\propto \exp\left(-\frac{\Delta G}{RT}\right)$$

is then enforced by adjusting $\left\{K_{i},K_{j}^{\prime }\right\}$ while maintaining the $\left\{[A_{i}]/K_{i},[P_{j}]/K_{j}^{\prime }\right\}$ ratio. Once the reaction parameters are fixed, one can execute equation (4.6) to reach steady states. The RT can be evaluated from the eigenvalues of the Jacobian matrix of a steady state as

4.11 $$\mathrm{RT}=-\frac{1}{N}\left(\sum_{i}\frac{1}{\lambda_{i}}\right).$$

The number of non-zero eigenvalues is determined by the rank of the stoichiometric matrix in equation (4.6). The stability of a solution is justified when all eigenvalues have negative real part. The RT values as a measure of the robustness of a given state are frequently referred to throughout this work. Furthermore, the efficiency of the state can be computed by the ratio of total carbon consumption to the biomass output.

Since only a subset of the complete metabolic reactions in a cell is selected for study, one can use a fictitious ‘External’ metabolite to represent the rest of a cell. A number of fictitious reactions connecting to the ‘External’ can be introduced. To specify a desired boundary condition, a bias flux *b_n_* can be set to/from the ‘External’ by modifying the corresponding $f_{n}(x,\;V)\to f_{n}(x,\;V)\mp b_{n}$ in equation (4.9). In a typical simulation, the outgoing fluxes to the External from a selected set of essential metabolites (biomass/energy output, see the non-zero ‘Biomass’ column in electronic supplementary material, table S5) are configured to match the physiological/growth scenario under consideration, whereas the fluxes on a handful of available nutrient intakes are self-regulated by the algorithm.

Note that equations (4.9)–(4.11), together with the boundary set-up contain the full set of mathematical equations necessary to carry out the numerical simulations. In a typical programming set-up, one will have a collection of metabolites and a large set of reactions between them, their numbers run into hundreds. A suitable initial set of {*V*_F_, *V*_B_} can be chosen in such a way that the reaction fluxes are kept small and uniformly distributed across the entire metabolic network. One then executes numerically the set of ordinary differential equations (ODEs) in equation (4.9) until a steady state of {*V*_F_, *V*_B_} is found. Once it is achieved, equation (4.11) is called to characterize the stability of the newly found state. The number of steady states so obtained is not unique. By varying the initial values of {*V*_F_, *V*_B_} for equation (4.9) a range of steady-state fluxes can be obtained and their properties can be analysed. Finally, simulations in the present study were carried out on Matlab platform with ode23t package as the core differential equation solver. Symbolic manipulation package was used in evaluating the eigenvalues in equation (4.11) on the steady states.

### 4.5. Sample preparation and RNA sequencing procedures

For total RNA extraction, cells in the early and late stationary phase at 16, 24, 36 and 72 h were rapidly cooled using chilled ethanol and harvested by centrifugation (12 000 r.p.m., 5 min, 4°C). Total RNA was extracted using RNeasy Mini Kit (Cat#74106, Qiagen) following the manufacturer's instructions and checked for an RIN number to inspect RNA integrity by an Agilent Bioanalyzer 2100 (Agilent technologies, Santa Clara, CA, US). Qualified total RNA was further purified by RNeasy micro kit (Cat#74004, QIAGEN, GmBH, Germany) and RNase-Free DNase Set (Cat#79254, QIAGEN, GmBH, Germany). RNA-sequencing was carried out on Illumina HiSeq 2500 by Shanghai Biotechnology Corporation (http://www.shbio.com/). After sequencing, clean RNA-Seq data were produced by eliminating the low-quality raw reads and mapping reads to the reference genome and gene sequences using SOAP2. The levels of gene expression were calculated using the RPKM method and those genes with FDR ≤ 0.001 and a log2 ratio greater than 1 were considered differentially expressed. Raw sequencing data have been deposited in the Arrayexpress database with accession number E-MTAB-7314.

## Supplementary Material

Supporting Information Figures

Reviewer comments

## Supplementary Material

Supporting Information Tables

## Data accessibility

Our transcriptome data in the manuscript have been deposited in Arrayexpress (http://www.ebi.ac.uk/arrayexpress) at the EBI under accession no. E-MTAB-7314. Dataset, the complete set of the reactions for the metabolic network, and selected initial/boundary conditions for eqns. (4.2) and (4.9) have been provided in electronic supplementary material.
