# Correction to: ‘Dynamical modelling of secondary metabolism and metabolic switches in *Streptomyces xiamenensis* 318’

**DOI:** 10.1098/rsos.190980

**Published:** 2019-06-19

**Authors:** Xiao-Mei Zhu, Xing-Xing Zhang, Run-Tan Cheng, He-Lin Yu, Ruo-Shi Yuan, Xu-Liang Bu, Jun Xu, Ping Ao, Yong-Cong Cheng, Min-Juan Xu

*R. Soc. open sci.* **6**, 190418. (Published 10 April 2019). (doi:10.1098/rsos.190418)

This correction refers to an error in the abstract. Transcriptome measurements were accidentally changed to ‘transcriptase measurements’. The correct text appears below.

Integrated studies via kinetic metabolic modelling, transcriptome measurements and metabolic profiling were performed on this strain.

