# Supplementary material for: Dynamical modelling of secondary metabolism and metabolic switches in Streptomyces xiamenensis 318
Source: R Soc Open Sci. 2019 Apr 10;6(4):190418. doi: 10.1098/rsos.190418 (PMC6502367; doi:10.1098/rsos.190418)
Supplement: Supporting Information Figures [file rsos190418supp1.docx]

**Dynamical modeling of secondary metabolism and metabolic switches in *Streptomyces xiamenensis* 318**

Xiao-Mei Zhu^†,‡,▽^, Xing-Xing Zhang^‡,▽^, Run-Tan Cheng^†^, He-Lin Yu^†^, Ruo-Shi Yuan^†^, Xu-Liang Bu^†,§^, Jun Xu^†^, Ping Ao^†,‡^, Yong-Cong Chen^‡^ and Min-Juan Xu^†^*

^†^ Key Laboratory of Systems Biomedicine (Ministry of Education), Shanghai Center for Systems Biomedicine, Shanghai Jiao Tong University, 800 Dongchuan Road, Shanghai 200240, P. R. China

^‡^ Shanghai Center for Quantitative Life Sciences & Physics Department, Shanghai University, Shanghai 200444, P. R. China

^§^ School of Oceanography, State Key Laboratory of Ocean Engineering, Shanghai Jiao Tong University, Shanghai 200240, P. R. China

[Figure S1. Schematic diagram of xiamenmycin biosynthesis and related central metabolic pathways in *S. xiamenensis* 318. 3](#_Toc524000804)

[Figure S2. Schematic diagram of biosynthesis and related metabolic pathways in S. xiamenensis 318. 4](#_Toc524000805)

[Figure S3. Enlarged metabolic network of S. xiamenensis 318 supporting both xiamenmycin and PTMs biosynthesis. 5](#_Toc524000806)

[Figure S4. Heatmap showing the two metabolic modes based on the normalized fluxes under different circumstances. 7](#_Toc524000807)

[Figure S5. Heatmap representing RNA-Seq transcriptome analysis for 25 selected enzyme genes in different pathways at a time series of 16 h, 24 h, 36 h and 72 h. 9](#_Toc524000808)

[Figure S6. Heatmap showing global profiles of calculated fluxes in the metabolic network 11](#_Toc524000809)

[Figure S7. Box plots summarize the distribution of raw calculated fluxes according to 129 reactions. Median values are shown (n = 158). The box represents the 25th and 75th percentiles. 13](#_Toc524000810)

[Table S1 14](#_Toc524000811)

[S1A The modeling results were obtained under growth condition, as shown below: (relaxation time: 228.75 s, initial value: 0.01) 14](#_Toc524000812)

[S1B The modeling results were obtained under growth condition, as shown below: (relaxation time: 197.11 s, initial value: 0.03) 14](#_Toc524000813)

[S1C The modeling results were obtained under growth condition, as shown below: (relaxation time: 157.49 s, initial value: 0.05) 14](#_Toc524000814)

[Table S2 14](#_Toc524000815)

[S2A The modeling results were obtained under growth condition, as shown below: (relaxation time: 197 s) 14](#_Toc524000816)

[S2B The modeling results were obtained under “knockout” condition of PTMs and xiamenmycin, as shown below: (relaxation time: 270 s) 14](#_Toc524000817)

[Table S3 15](#_Toc524000818)

[S3A The modeling results were obtained under limit growth condition of low PTM yied, as shown below: 15](#_Toc524000819)

[S3B The modeling results were obtained under limit growth condition of high PTM yied, as shown below: 15](#_Toc524000820)

[Table S4 The modelling results of metabolic reaction at time series samples of S. xiamenensis 318 15](#_Toc524000821)


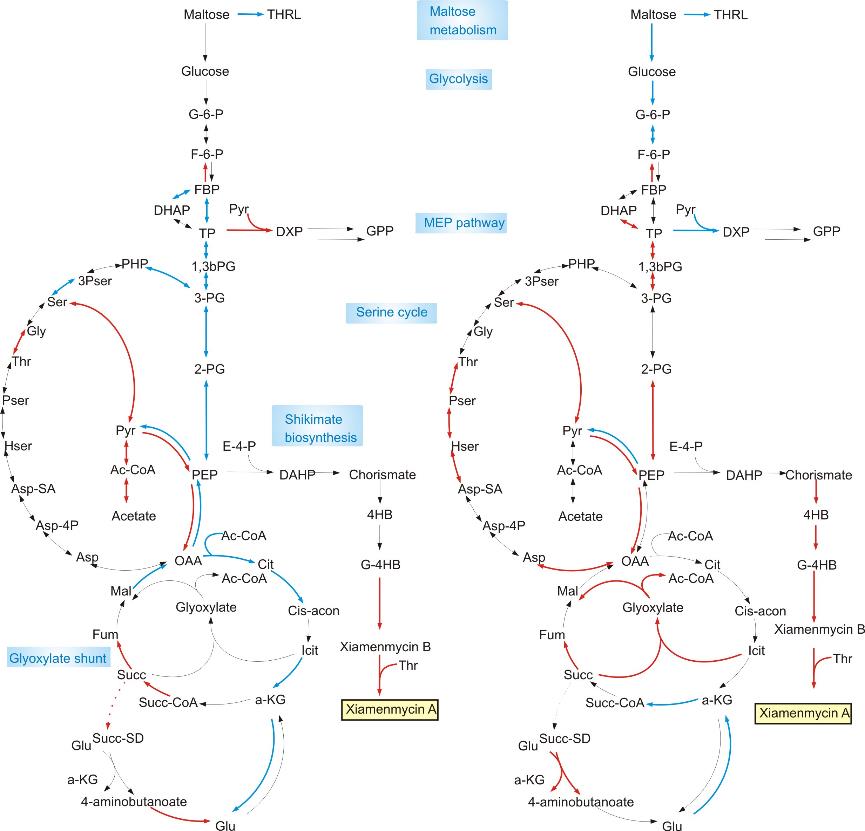


Fig. S1. Schematic diagram of xiamenmycin biosynthesis and related central metabolic pathways in *S. xiamenensis* 318. The figure shows the up-regulated gene expressions (in red) and down-regulated gene expressions (in blue) at different time points. A) 16 h vs 36 h; B) 36 h vs 72 h.

# Figure S1. Schematic diagram of xiamenmycin biosynthesis and related central metabolic pathways in *S. xiamenensis* 318.

The figure shows the up-regulated gene expressions (in red) and down-regulated gene expressions (in blue) at different time points. A) 16 h vs 36 h; B) 36 h vs 72 h.


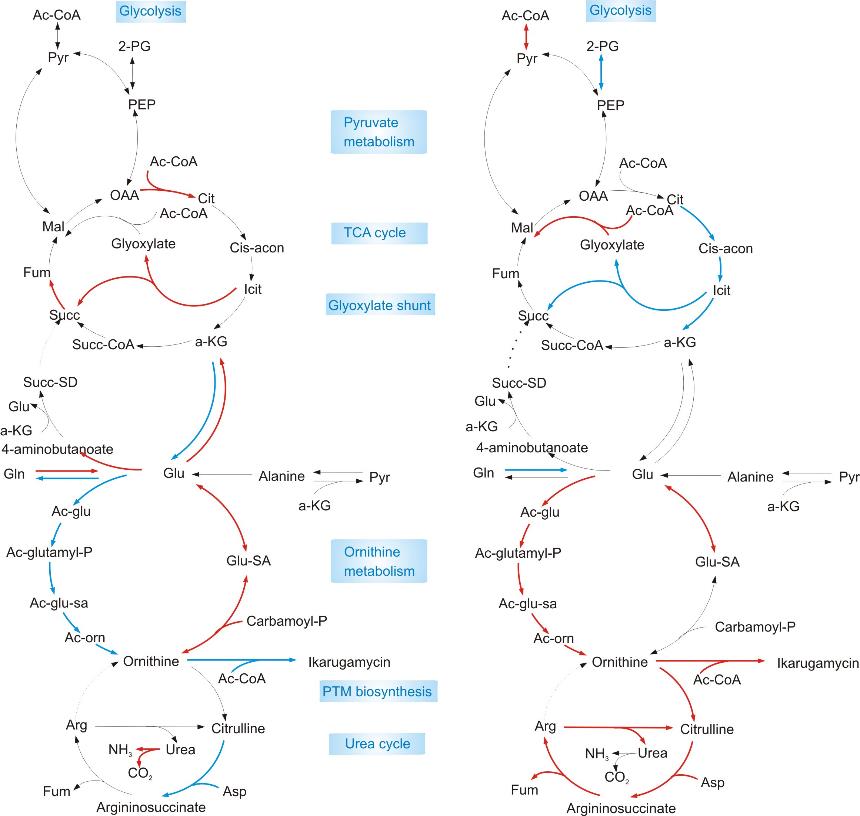


# Figure S2. Schematic diagram of biosynthesis and related metabolic pathways in S. xiamenensis 318.

The figure shows the up-regulated gene expressions (in red) and down-regulated gene expressions (in blue) at different time points. A) 16 h vs 24 h; B) 24 h vs 36 h.


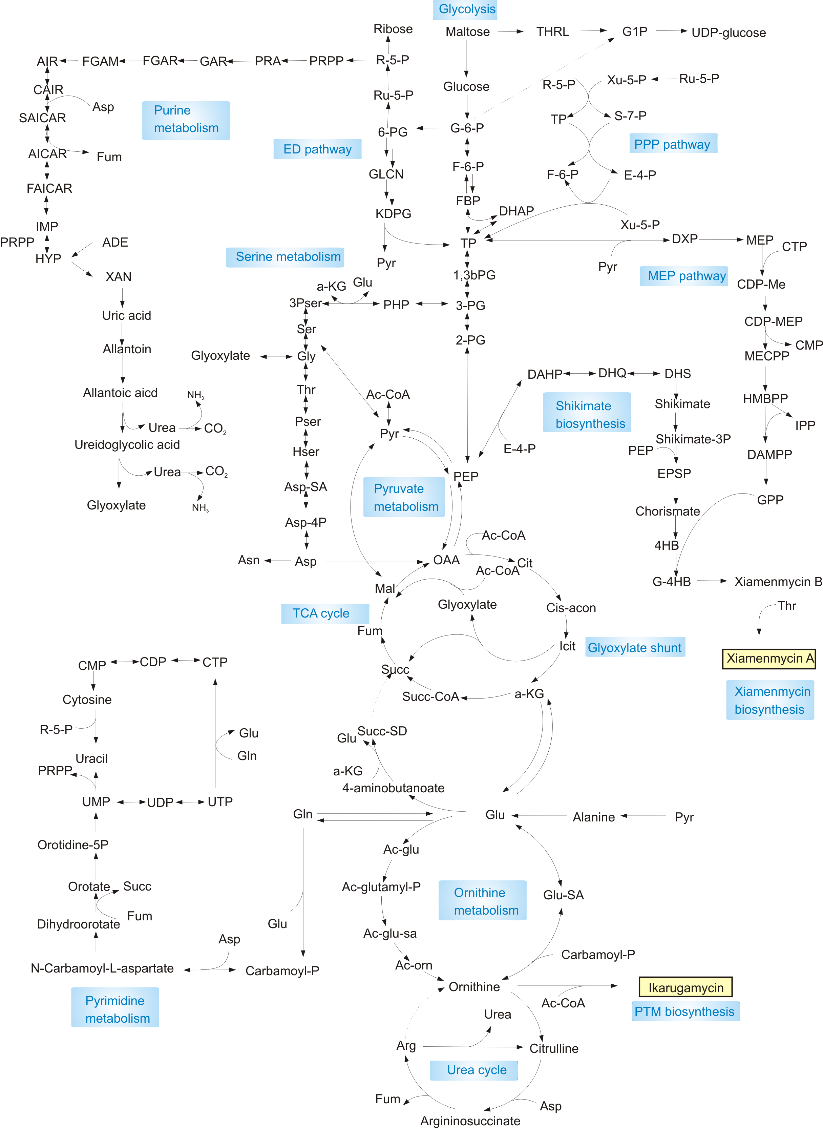


# Figure S3. Enlarged metabolic network of S. xiamenensis 318 supporting both xiamenmycin and PTMs biosynthesis.

The central carbon metabolism for *S. xiamenensis* 318 is illustrated here by arrows and includes glycolysis/gluconeogenesis, citric acid cycle, pyruvate metabolism, pentose phosphate pathway, glyoxylate shunt, glycine-serine-threonine metabolism, ornithine metabolism, purine and pyrimidine metabolism, urea cycle and shikimate biosynthesis, as well as MEP biosynthesis. Biosynthesis of xiamenmycin and PTMs in *S. xiamenensis* 318 have also been mapped.


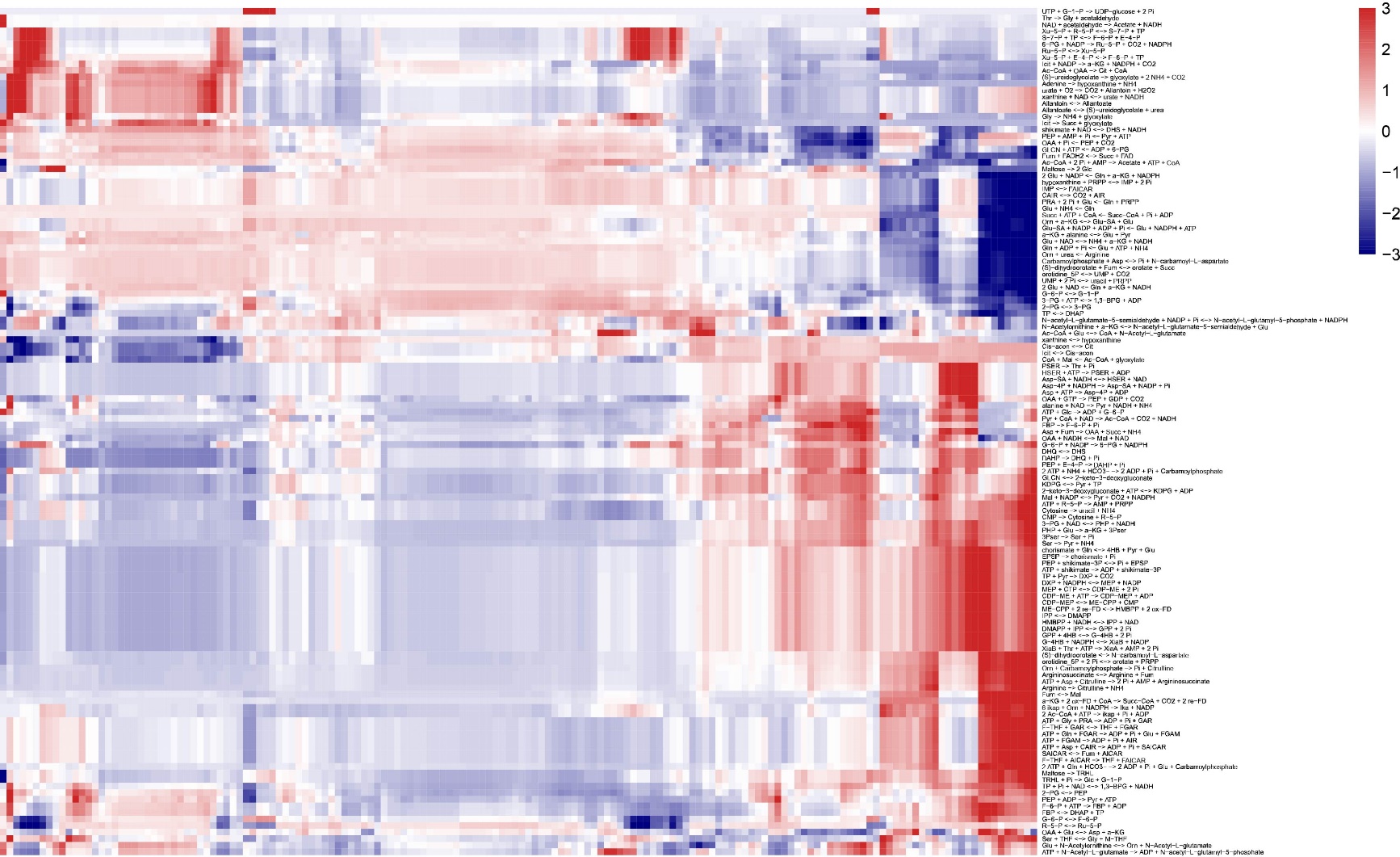


# Figure S4. Heatmap showing the two metabolic modes based on the normalized fluxes under different circumstances.

The flux profiles of pathways in the kinetic metabolic model of Streptomyces xiamenensis 318 after 158 times calculation. A total of 129 reactions are included.


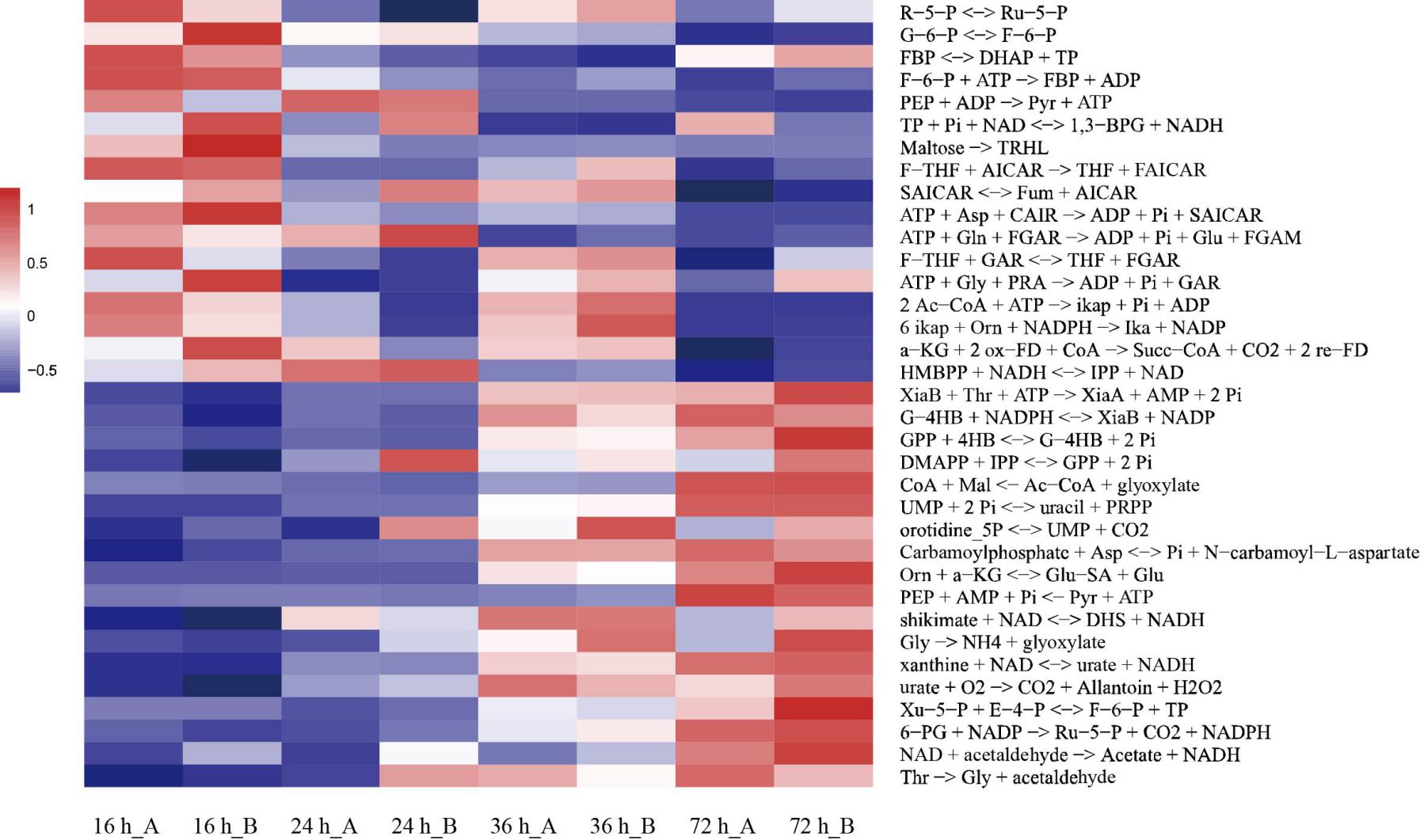


# Figure S5. Heatmap representing RNA-Seq transcriptome analysis for 25 selected enzyme genes in different pathways at a time series of 16 h, 24 h, 36 h and 72 h.


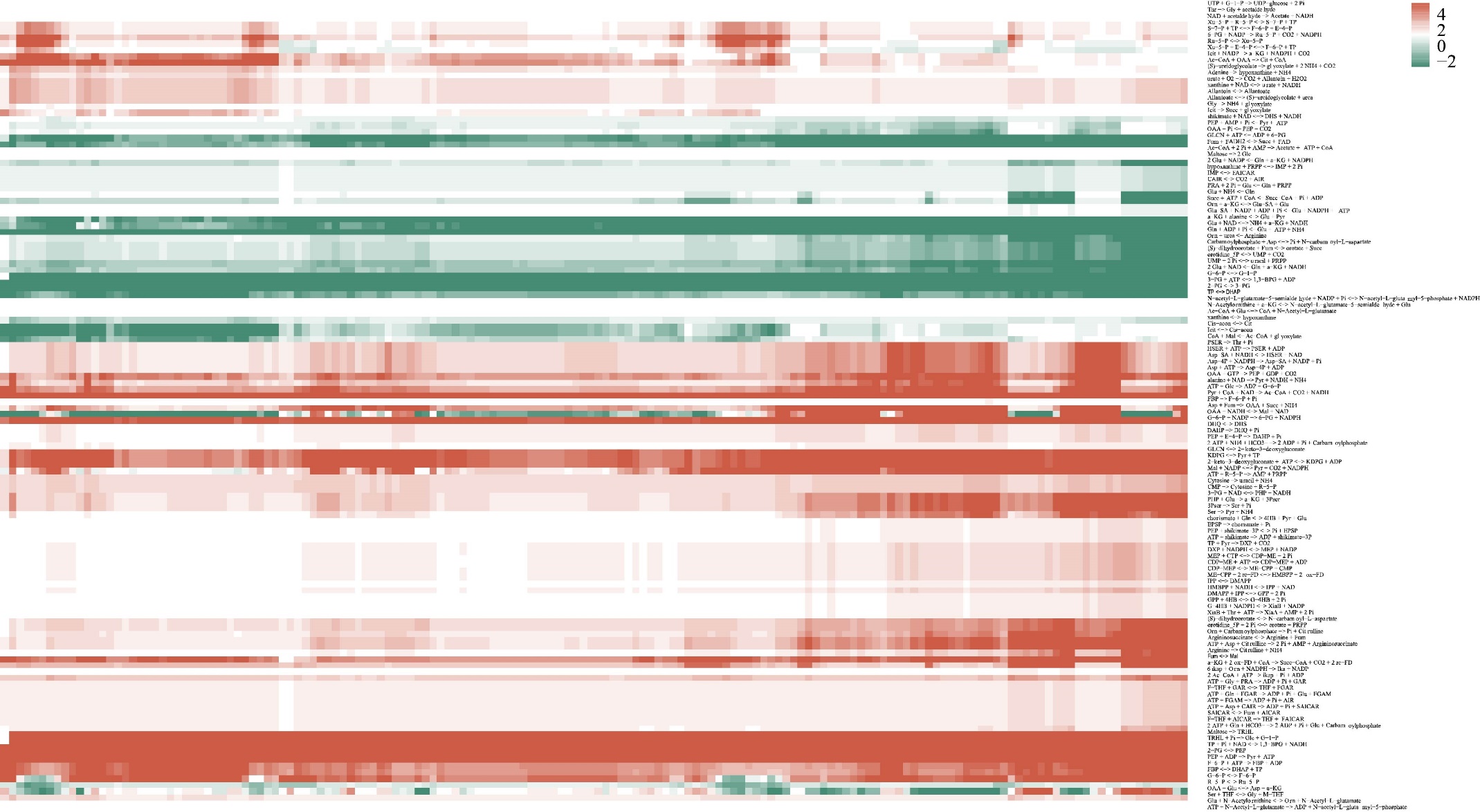


# Figure S6. Heatmap showing global profiles of calculated fluxes in the metabolic network


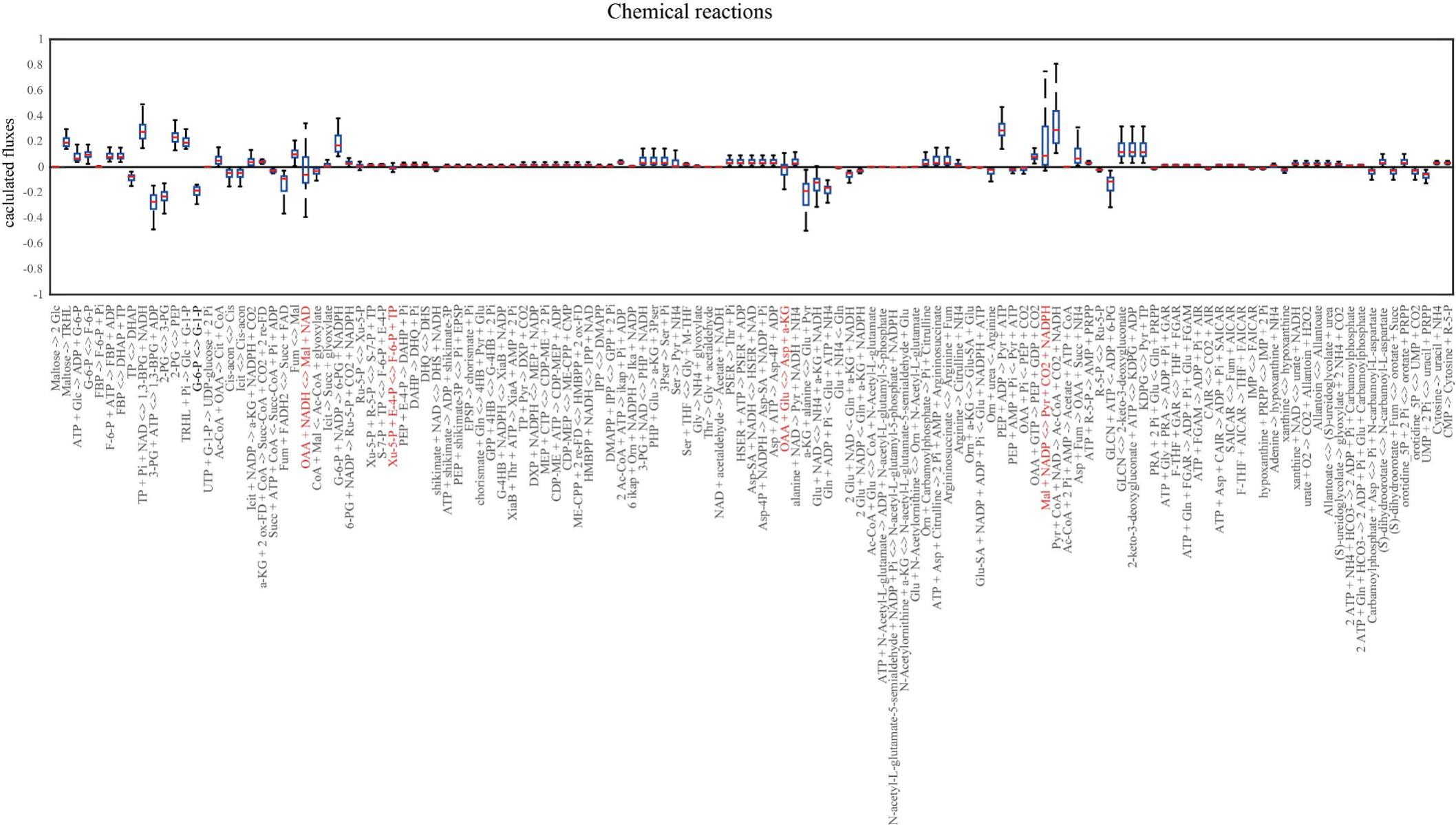


# Figure S7. Box plots summarize the distribution of raw calculated fluxes according to 129 reactions. Median values are shown (n = 158). The box represents the 25th and 75th percentiles.

# Table S1

# S1A The modeling results were obtained under growth condition (relaxation time: 228.75 s, initial value: 0.01)

# S1B The modeling results were obtained under growth condition (relaxation time: 197.11 s, initial value: 0.03)

# S1C The modeling results were obtained under growth condition (relaxation time: 157.49 s, initial value: 0.05)

# Table S2

# S2A The modeling results were obtained under growth condition (relaxation time: 197 s)

# S2B The modeling results were obtained under “knockout” condition of PTMs and xiamenmycin (relaxation time: 270 s)

# Table S3

# S3A The modeling results were obtained under limit growth condition of low PTM yield

# S3B The modeling results were obtained under limit growth condition of high PTM yield

# Table S4 The modelling results of metabolic reactions at time series samples of S. xiamenensis 318

# Table S5 The list of metabolites used in the simulations for Table S1A. Note that non-zero values in the “Biomass” column are used to specify the flux-in or flux-out boundary conditions for the metabolic network.
