# Supplementary material for: Dynamical modelling of secondary metabolism and metabolic switches in Streptomyces xiamenensis 318
Source: R Soc Open Sci. 2019 Apr 10;6(4):190418. doi: 10.1098/rsos.190418 (PMC6502367; doi:10.1098/rsos.190418)
Supplement: Reviewer comments [file rsos190418_review_history.pdf]

THE ROYAL SOCIETY
PUBLISHING

ROYAL SOCIETY
OPEN SCIENCE

Dynamical modelling of secondary metabolism and metabolic switches in *Streptomyces xiamenensis* 318

Xiao-Mei Zhu, Xing-Xing Zhang, Run-Tan Cheng, He-Lin Yu, Ruo-Shi Yuan, Xu-Liang Bu, Jun Xu, Ping Ao, Yong-Cong Chen and Min-Juan Xu

Article citation details
R. Soc. open sci. **6**: 190418.
http://dx.doi.org/10.1098/rsos.190418

Review timeline
Original submission: 13 March 2019
Final acceptance: 18 March 2019

Note: Reports are unedited and appear as submitted by the referee. The review history appears in chronological order.

Note: This manuscript was transferred from another Royal Society journal with peer review.

Review History

Decision letter (RSOS-190418)

18-Mar-2019

Dear Dr Xu,

I am pleased to inform you that your manuscript entitled "Dynamical modeling of secondary metabolism and metabolic switches in Streptomyces xiamenensis 318" is now accepted for publication in Royal Society Open Science.

You can expect to receive a proof of your article in the near future. Please contact the editorial office (openscience_proofs@royalsociety.org and openscience@royalsociety.org) to let us know if you are likely to be away from e-mail contact. Due to rapid publication and an extremely tight schedule, if comments are not received, your paper may experience a delay in publication.

Royal Society Open Science operates under a continuous publication model (http://bit.ly/cpFAQ). Your article will be published straight into the next open issue and this will be the final version of the paper. As such, it can be cited immediately by other researchers.

Reports © 2019 The Reviewers; Decision Letters © 2019 The Reviewers and Editors; Responses © 2019 The Reviewers, Editors and Authors. Published by the Royal Society under the terms of the Creative Commons Attribution License http://creativecommons.org/licenses/by/4.0/, which permits unrestricted use, provided the original author and source are credited

As the issue version of your paper will be the only version to be published I would advise you to check your proofs thoroughly as changes cannot be made once the paper is published.

On behalf of the Editors of Royal Society Open Science, we look forward to your continued contributions to the Journal.

Kind regards,
Royal Society Open Science Editorial Office
Royal Society Open Science
openscience@royalsociety.org

on behalf of Professor Pietro Cicuta (Subject Editor)
openscience@royalsociety.org

Follow Royal Society Publishing on Twitter: @RSocPublishing
Follow Royal Society Publishing on Facebook:
https://www.facebook.com/RoyalSocietyPublishing.FanPage/
Read Royal Society Publishing's blog: https://blogs.royalsociety.org/publishing/
